# Development of a new AgriSeq 4K mid-density SNP genotyping panel and its utility in pearl millet breeding

**DOI:** 10.3389/fpls.2022.1068883

**Published:** 2023-01-10

**Authors:** Janani Semalaiyappan, Sivasubramani Selvanayagam, Abhishek Rathore, SK. Gupta, Animikha Chakraborty, Krishna Reddy Gujjula, Suren Haktan, Aswini Viswanath, Renuka Malipatil, Priya Shah, Mahalingam Govindaraj, John Carlos Ignacio, Sanjana Reddy, Ashok Kumar Singh, Nepolean Thirunavukkarasu

**Affiliations:** ^1^ Genomics and Molecular Breeding Lab, ICAR-Indian Institute of Millets Research, Rajendranagar, India; ^2^ Accelerated Crop Improvement, International Crop Research Institute for the Semi-Arid Tropics (ICRISAT), Hyderabad, India; ^3^ Excellence in Breeding (EiB) Platform, The International Maize and Wheat Improvement Center (CIMMYT), El Batán, Mexico; ^4^ Bioinformatics, Thermo Fisher Scientific, Austin, TX, United States; ^5^ HarvestPlus, International Center for Tropical Agriculture, Cali, Colombia; ^6^ Department of Horticulture and Crop Science, The Ohio State University, Wooster, OH, United States; ^7^ ICAR-Indian Agricultural Research Institute, New Delhi, India

**Keywords:** pearl millet, mid-density SNP, AgriSeq technology, high-throughput genotyping, genomics

## Abstract

Pearl millet is a crucial nutrient-rich staple food in Asia and Africa and adapted to the climate of semi-arid topics. Since the genomic resources in pearl millet are very limited, we have developed a brand-new mid-density 4K SNP panel and demonstrated its utility in genetic studies. A set of 4K SNPs were mined from 925 whole-genome sequences through a comprehensive in-silico pipeline. Three hundred and seventy-three genetically diverse pearl millet inbreds were genotyped using the newly-developed 4K SNPs through the AgriSeq Targeted Genotyping by Sequencing technology. The 4K SNPs were uniformly distributed across the pearl millet genome and showed considerable polymorphism information content (0.23), genetic diversity (0.29), expected heterozygosity (0.29), and observed heterozygosity (0.03). The SNP panel successfully differentiated the accessions into two major groups, namely B and R lines, through genetic diversity, PCA, and structure models as per their pedigree. The linkage disequilibrium (LD) analysis showed Chr3 had higher LD regions while Chr1 and Chr2 had more low LD regions. The genetic divergence between the B- and R-line populations was 13%, and within the sub-population variability was 87%. In this experiment, we have mined 4K SNPs and optimized the genotyping protocol through AgriSeq technology for routine use, which is cost-effective, fast, and highly reproducible. The newly developed 4K mid-density SNP panel will be useful in genomics and molecular breeding experiments such as assessing the genetic diversity, trait mapping, backcross breeding, and genomic selection in pearl millet.

## Introduction

Pearl millet (*Pennisetum glaucum* (L) R. Br., syn. *Cenchrus americanus* (L.) Morrone) is a strategic climate-resilient C4 crop. It has an inherent ability to provide sustainable yield even in harsh ecologies, making it an economically secure and favorable crop for farmers in semi-arid and arid regions of the world. Pearl millet is also known for nutritional security as it is competent to address malnutrition issues (Nambiar et al., 2011; Kanatti et al., 2014). In traditional plant breeding, superior genotypes have been selected visually. With the discovery of genetic markers, crop breeding heavily depends upon the reliable and cost-effective marker system. Developing viable markers and genotyping platforms in any crop is imperative to accelerate the varietal turnover.

Various marker genotyping methods such as expressed sequence tags- derived simple sequence repeats (EST-SSRs) (Senthilvel et al., 2008; Rajaram et al., 2013), genomic simple sequence repeats (gSSRs), (Qi et al., 2004), DArT array Technology (DArTs) (Senthilvel et al., 2010; Supriya et al., 2011), and single nucleotide polymorphisms (SNPs) (Sehgal et al., 2012) have been developed and used to characterize the pearl millet genome, identification of quantitative trait loci (QTLs) and marker-assisted breeding (MAB)

activities. The EST-SSRs were developed and utilized in the genetic mapping and MAB programs targeting the traits such as yield and drought resistance in pearl millet (Senthilvel et al., 2010). Through DArT and SSRs marker systems, the QTLs for grain iron and zinc content and rust resistance were identified in the RILs population of pearl millet (Ambawat et al., 2016; Kumar et al., 2016; Kumar et al., 2018). Later, the EST-derived SNPs markers were developed in pearl millet and deployed for identifying the major QTLs-associated candidate genes for drought tolerance using two mapping populations (Sehgal et al., 2012). The *de-novo* sequencing of the pearl millet genome (1.79 Gb), (Varshney et al., 2017) provided an opportunity to explore the genome comprehensively and develop new genomic resources. They have a great potential for understanding the genetic architecture and quantitative traits as well as improving such traits in pearl millet.

There are mainly three types of throughput platforms, namely high-density (10’s of thousands of SNPs), mid-density (a few thousand SNPs), and low-density (less than 100 SNPs), generally used for genotyping purposes. The high-density platforms are usually applicable for whole genome studies and trait mapping but are very tedious, expensive, laborious, and time-consuming. High-density platforms such as Illumina, PacBio, genotyping-by-sequencing (GBS), and restriction site-associated DNA sequencing (RAD) provide tens of thousands of genome-wide SNPs. The mid-density platforms, which include AgriSeq (Koelewijn, 2018), DartTag (Kilian et al., 2012; Ren et al., 2015), and RiCA (Arbelaez et al., 2019) have their applicability in genotyping 1000 to 5000 SNPs and are highly advantageous in terms of being time-efficient and user-friendly with rapid data interpretation. Low-density platforms are mainly used to track specific QTLs or genes. TaqMan and KASP™ (Kompetitive Allele-Specific Polymerase chain reaction) are the widely used low-density SNP platforms (Thomson et al., 2012; Ganal et al., 2019). Array-based SNP chips are developed in several crops by including the identified SNPs printed on the chip. For example, in maize, Illumina developed a golden gate assay Illumina^®^ 1536 SNP chip and Illumina^®^ MaizeSNP50 Beadchip (www.illumina.com/maizeSNP50, Wu et al., 2014) and has been used for various genetic applications (Thirunavukkarasu et al., 2013; Nepolean et al., 2014; Thirunavukkarasu et al., 2017).

The mid-density genotyping approach is highly efficient, informative, and cost-effective as it can be used in various genomics and molecular breeding experiments such as diversity assessment, trait mapping, marker-assisted breeding, and genomic selection. Although a crop with immense economic and social importance, pearl millet has been neglected for a long time, and not enough efforts have been made to explore genomic resources. Hence, the objectives of the experiment were to mine a mid-density panel of 4000 SNPs from 925 whole genome sequences of pearl millet, to develop a functional, robust, and reproducible genotyping protocol through AgriSeq technology, to characterize the newly developed SNPs in a set of 373 genetically diverse pearl millet B and R lines and to demonstrate its utility in genetic studies.

## Material and methods

### Mining SNPs

The whole-genome resequences (WGRS) from two sets of pearl millet accessions (Varshney et al., 2017) were considered for developing the mdi-density marker panel− 1. The Pearl Millet Inbred Germplasm Association Panel (PMiGAP) representing 345 genotypes (263 landraces or traditional cultivars, 46 breeding lines, 25 advanced or improved cultivars, and 11 accessions with unknown biological status) (hereafter “Group A” genotypes) and 2. Diverse breeding lines representing 580 genotypes (260 B and 320 R lines) (hereafter “Group B” genotypes), assuming to accommodate possible racial and geographical representation of pearl millet breeding diversity.

#### Extraction of SNPs from the Group A and B genotypes

**Figure 1:**
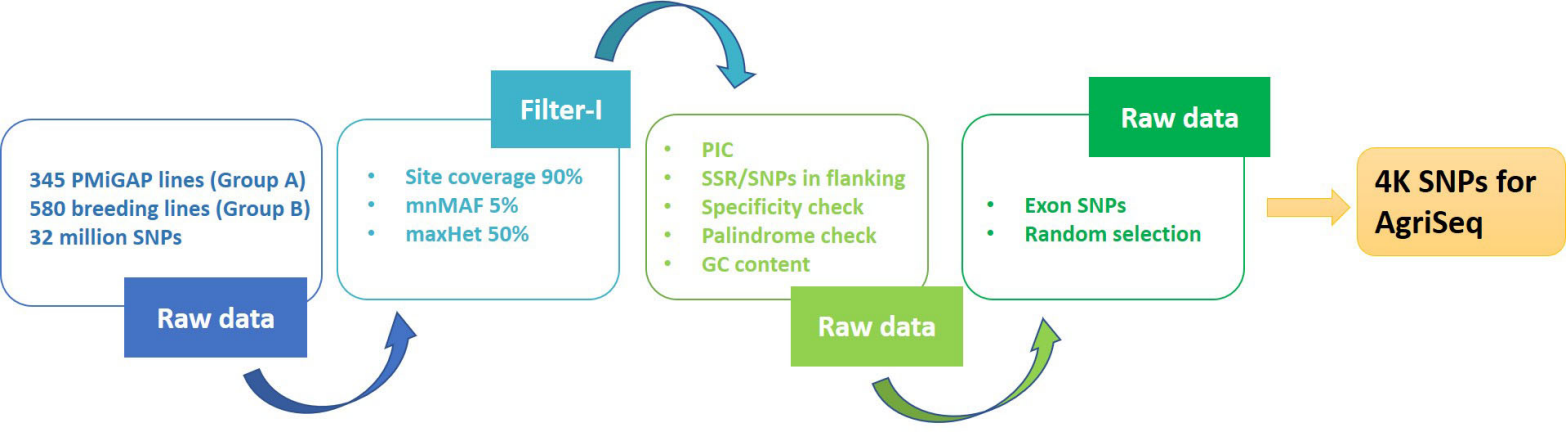
*In-silico* pipeline used in extraction of 4K mid-density SNPs from the 925 genetically diverse pearl millet genome sequences.

The variant data (32 million SNPs) from the Groups A and B genotypes were retrieved from the pearl millet genome project (Varshney et al., 2017). These variants were pruned for site coverage (90%) and minimum minor allele frequency (0.01) using Tassel (version 5.2.51), which resulted in 276K bi-allelic SNP markers. Filtration of the markers mentioned above for specificity, polymorphic information content (PIC), flanking SNPs on windows (50bp), and presence of flanking SSRs (with pearl millet reference genome v1.1) resulted in 67K SNPs. For the specificity check, we used Bowtie (v1.1.2) with no variations allowed upon mapping. Only the SNPs falling on exons were taken forward from the filtered variants. This marker set was further put on two independent random selection exercises, using the purity tool (https://bitbucket.org/jcignacio/purity/wiki/Home), for picking an initial set of 6000 markers each that can carry maximum genetic information. These two sets (from different iterations) filtered for high LD (LDBlockShow) and redundant markers, resulting in 2000 SNPs each for Group A (2K set I) and B genotypes (2K set II), respectively. The 2K sets I and II together formed a 4K mid-density SNP panel (4K set). The 4K panel were functionally annotated using SnpEff (v4.3t) with predicted pearl millet reference annotations (**Figure 1**).

### SNP assay design

The selected SNPs were passed through AgriSeq’s design quality control process. The quality check was performed using the pearl millet reference genome (accession: GCA_002174835.2) and then submitted to the primer design phase. The primer designs were *in-silico* checked for specificity and sensitivity of the intended target/marker regions using pearl millet reference genome. Finally, 4000 SNPs were selected to constitute the custom 4K SNP GBS pearl millet panel.

#### Sequencing

The AgriSeq targeted-GBS solution utilizes a highly efficient multiplexed PCR chemistry where hundreds to thousands of markers can be targeted and uniformly amplified in a single reaction. Three eighty-four samples were prepared for sequencing using the AgriSeq HTS Library Kit (A34143-Life Technologies). In short, DNA concentrations were normalized to 3.3 ng/µL for a total of 10 ng DNA per 10 µL reaction. Normalized DNA was combined with the AgriSeq custom primer panel and AgriSeq amplification master mix. For amplification of genomic targets, the following thermocycling programs were used; 99°C for 2 minutes, then 15 cycles of 99°C for 15s and 60°C for 4 minutes. Amplicons were prepared for ligation with pre-ligation enzyme digestion at 50°C for 10 minutes, 55°C for 10 minutes, and 60°C for 20 minutes. IonCode™ Barcode Adapters 385-768 Kit (A36546-Life Technologies) were ligated to the digested products with barcoding enzyme and buffer. Labeled amplicons were then pooled, cleaned up, amplified, and normalized. Following library preparation, libraries were loaded onto an Ion 540™ sequencing Chip Kit (A42849) *via* the Ion 540™ Kit-Chef (A43541-Life Technologies) and Ion Chef. Sequencing was performed on the Ion S5 system (Thermo Fisher, Inc. Waltham, MA). After sequencing, genotyping was performed automatically by Torrent Variant Caller (TVC) on the Torrent Suite Server (TS).

#### Genotyping

The variant calling pipeline is fully automated and optimized for analyzing Ion Torrent sequencing data on Torrent Suite Server (Thermo Fisher Scientific). This workflow comprises several series of steps. First, signal processing files are automatically transferred from the sequencing platform to the S5 server and then converted to raw reads (FASTQ). After that, the sequenced reads were de-multiplexed to individual samples using the barcode sequences. For each sample, the sequenced reads from the targeted regions were mapped to the pearl millet reference genome using TMAP- Torrent Mapping Alignment Program (https://github.com/iontorrent/TS/tree/master/Analysis/TMAP) followed by genotyping using TVC-Torrent Variant Caller (https://github.com/iontorrent/Torrent-Variant-Caller-stable). The genotypes were reported in different formats TOP, TOP/BOT, and actual alleles using AgriSum Toolkit, an AgriSeq TS plugin (https://assets.thermofisher.com/TFS-assets/LSG/manuals/MAN0018917_AgriSum_plugin_UB.pdf).

### Data analysis

#### SNP statistics

A set of 373 pearl millet inbreds consisting of 195 B-lines and 182 R-lines received from ICRISAT, Hyderabad (**Supplementary Table S1**) were subjected to genotyping using the newly developed 4K SNP panel. The genotyping data were analyzed for several metrics, namely PIC, Nei’s genetic diversity (GD), minor allele frequency (MAF), expected heterozygosity (He), and observed heterozygosity (Ho). The parameters mentioned above were calculated using the SnpReady package in R (Granato et al., 2018).

#### Principal component analysis

PCA was conducted using the snpgdsPCA function available in SNPRelate (Zheng et al., 2012). The percentage of variation was calculated for the first 15 principal components, and the genotypes were plotted on a three-dimensional scale using the first three components.

#### Analysis of molecular variance

The variance at the molecular level of 373 genotypes between and within B- and R-line groups was analyzed through GENEALEX version 6.503 (Peakall and Smouse, 2006) with 999 permutations of the data set using PhiPT value (an analog of fixation index FST). PhiPT in AMOVA, a measure that provides significant insights into the evolutionary processes that influence the structure of genetic variation within and among subgroups, was used to calculate the degree of genetic divergence. PhiPT value represents the ratio of the variance within subgroups to the overall variance between subgroups (Keneni et al., 2012). The high PhiPT value indicates the more significant differences between the subgroups.

#### Diversity assessment

DARwin (version 6.0.9) (Perrier and Jacquemoud-Collet, 2006) was used for measuring the genetic diversity among the accessions. The unweighted neighbor-joining approach was used to visualize the phylogenetic tree from the dissimilarity coefficient based on a simple matching approach.

#### Linkage disequilibrium

The extent of the LD in the 373 genotypes in all three set SNP markers was evaluated using TASSEL 5 (Bradbury et al., 2007). For each pair of SNP markers, the squared correlation coefficient (r^2^), which measures the correlation between alleles at two loci, was computed along with its corresponding P-value. The SNPs with all MAF, 15% heterozygotes, and 20% missing were included in this analysis. LD values of all pair-wise SNPs were shown in triangle LD plots using TASSEL the genome-wide and chromosome-wise LD patterns.

#### Population structure

The population structure of the accessions was estimated using an MCMC (Markov Chain Monte Carlo) model implemented in STRUCTURE version 2.3.4 (Pritchard et al., 2000). The data set was evaluated for each K value (2 to 10) with five iterations. The burn-in and MCMC replication numbers were set to 200000 for each run. The most probable K value was determined using the log probability of the data [LnP(D)] and delta K(ΔK) in Structure Harvester (Earl and Vonholdt, 2012). After the optimum K was determined, the graphical representation of the population structure was displayed using the CLUMPACK beta version (Kopelman et al., 2015).

## Results

### Basic statistics

For the preliminary statistics, the genotypic data from the two SNP sets, 2K set I and 2K set II, were compared since they were derived from two different sets of genotypes. Then, the combined genotypic data, the 4K set, was used for the remaining statistical studies. The data were used to calculate the marker call rate, i.e., the percentage of samples for a particular marker that generate a genotype call. The mean and the median marker call rate for the 2K set I was 93.8% and 98.9%, respectively, while it was 82.9% and 98.9%, respectively, for the 2K set II. For the combined 4K Set, the mean and the median marker call rate was 88.2% and 98.1%, respectively.

**Figure 2:**
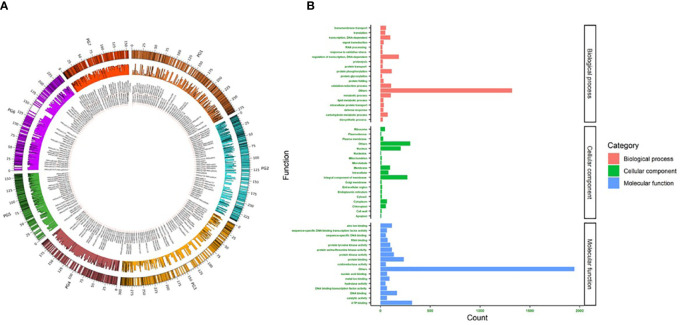
**(A)** Circos plot showing the location and uniform distribution of 4K mid-density SNPs (outer circle), SNP IDs (inner most circle), and PIC values (middle circle) across pearl millet chromosomes. **(B)** Gene ontology of 4K SNPs classified as biological process, cellular component and molecular function.

In 2K sets I and II, most SNPs were located on chromosomes 2, 1, and 3. Chr2 had comparatively more SNPs in both sets (set I-17.9% and set II-18.8%) (**Figure 2**). The major allele frequency ranged from 0.5 to 0.99 in both sets, while set I had a higher mean frequency (0.86) over set II (0.73). On the other hand, higher MAF was observed in set II (0.2) over set I (0.14). In set I, 815 detected SNPs was involved in various biological processes contributing to 40.8% of total SNPs, while 31.3% (625) and 43.4% (868) of SNPs were involved in molecular function and cellular components, respectively. Set II showed a similar number of SNPs (832, 39.39%) involved in various biological processes. SNPs involved in molecular function were higher (1137, 53.83%), and cellular components were lesser (344, 16.28%) when compared to Set I (**Figure 2**). The SNPs identified in the downstream region were 645 and 685, upstream regions were 482 and 651, exon regions were 237 and 293, and intron regions were 636 and 483 for sets I and II, respectively.

The PIC, Ho, and He values were determined using the SNPReady for the data 2K set I, 2K set II, and combined 4K set (**Table 1**). The PIC for both the sets, 2K set I and II, ranged from 0 to 0.38, while the mean PIC was high in 2K set II (0.3). When comparing sets I and II, 62% of the SNPs showed more than average PIC in 2K set II. About 58% from set II and 44% of SNPs from the 4K set possessed a PIC value that was more than the average. R lines of 2K set II showed the highest mean PIC of 0.28 among all sets, while B-lines showed the highest PIC value (0.27) in the same data set.

**Table 1:** Characteristics of the 2K Set I, 2K Set II and 4K Set of SNPs genotyped in a set of 373 B- and R-lines of pearl millet.

	Description	2K Set I	2K Set II	4K Set
PIC	Overall range	0 - 0.38	0 - 0.38	0 - 0.38
	Mean	0.16	0.3	0.24
	B-line mean	0.14	0.27	0.21
	R line mean	0.16	0.28	0.23
Ho	Overall range	0-0.21	0-0.41	0-0.3
	Mean	0.02	0.04	0.03
	B-line mean	0.02	0.05	0.04
	R-line mean	0.02	0.03	0.02
He	Overall range	0-0.21	0-0.38	0-0.31
	Mean	0.19	0.38	0.29
	B-line mean	0.16	0.34	0.25
	R-line mean	0.19	0.35	0.27

PIC-Polymorphic information content, Ho- Observed heterozygosity, He- Expected heterozygosity.

The observed heterozygosity (Ho) in 2K set I and 2K set II and 4K set ranged from 0 to 0.21, 0 to 0.41 and 0 to 0.31, while the expected heterozygosity (He) ranged from 0 to 0.21, 0 to 0.38 and 0to 0.3, respectively. 2K set II showed the highest average observed and expected heterozygosity (Ho= 0.04 and He= 0.38). When comparing the B- and R-lines groups, the B-lines had more average Ho in 2K set II and 4K set than the R-lines, whereas the R lines had more average He over the B-lines among all three data sets (**Table 1**).

### Genetic diversity, PCA, and population structure analysis

The grouping behavior of B and R lines was characterized using the 4K set data through genetic diversity, principal component analysis, and structure models.

**Figure 3:**
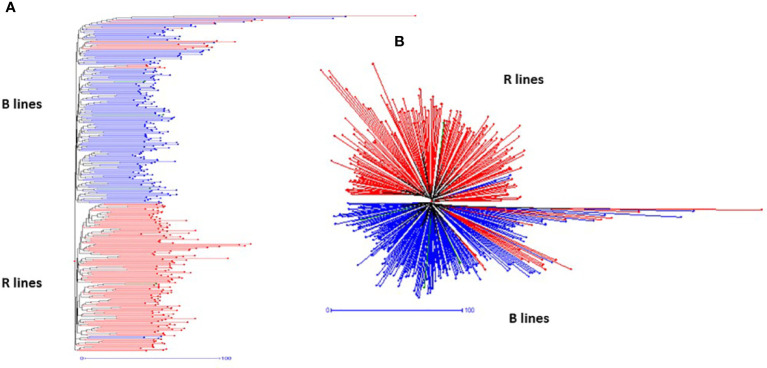
Hierarchical **(A)** and radial **(B)** topologies showing the clustering pattern of 373 pearl millet lines based on 4K SNP data.

The SNP frequency-based genetic dissimilarity matrix available in DARwin-6.0 was employed to study the genetic diversity among the 373 genotypes, which included 195 B- and 182-R lines. The NJ-based statistics grouped all the B- and R-lines into two clear-cut major groups (**Figure 3**) with additional sub-clusters in the respective groups. The first major group (G-I) consisted of 191 B- and 21 R-lines and the second major group (G-II) consisted of 155 R- and 4 B-lines. We also found that one set of R-lines (6 genotypes) formed a small third group. The G-I was further separated into three sub-groups (SG-IA, SG-IA, and SG-IC). SG-IA had 167 B- and 21 R-lines, while SG-IB and SG-IC had 11 and 13 B-lines, respectively. The second major group G-II further grouped the R lines into three subgroups. SG-IIA had 147 R-lines and one B-line, SG-IIB consisted of 2 R-lines and 3 B-lines, and SG-IIC formed by 6 R-lines.

We have also noticed cross-grouping of genotypes, where the R-lines, namely, ICMR 100258, ICMR 102545, ICMR 102497, ICMR 102499, ICMR 11555, ICMR 14111, ICMR 16444, ICMR 16333, ICMR 19888, ICMR 15111, ICMR 07333, ICMR 0755, ICMR 10111, ICMR 13666, ICMR 11888, ICMR 10222, ICMR 07666 and ICMR 15333 grouped with B- line clusters and B lines, ICMB 101839, ICMB 101912, ICMB 1502 and ICMA1 19888 grouped with R-line clusters (**Figure 3**).

The average mean Nei’s genetic diversity (GD) among all 373 genotypes was 0.29. Comparing the B- and R-lines, the mean genetic diversity (0.28) of the R lines was higher than the B lines (0.26). Here, 57% of SNPs covered more than the mean GD value among all accessions.

The pair-wise genetic dissimilarity analysis showed that the dissimilarity coefficient for the entire population ranged from 0.003 to 0.68. The minimum and maximum genetic dissimilarly between B- and R-line groups were 0.006 and 0.68, respectively. Within B- and R-line groups, the R-line pairs showed higher genetic dissimilarity (maximum 0.66) over the B-line pairs (maximum 0.58). The total dissimilarity measured in the population was classified into low (0.00 to 0.25), medium (0.25 to 0.50), and high (>0.50) to understand the frequency of pairs present in the respective dissimilarity group. It was observed that 817, 36, and 228 pairs from the B × R, B × B, and R × R groups fell in the high category. On the other hand, 158, 2431, and 1766 pairs were identified as having low genetic dissimilarity under the B × R, B × B, and R × R groups, respectively. It explained that the diversity among the R × R lines was much higher than the B × B lines.

In the B × R group, genotypes namely, ICMR 100258, ICMR 16333, ICMR 102277, ICMA1 101813, ICMA1 101805, and ICMA1 1803 showed significant genetic diversity over others as they frequently occurred under the high-dissimilarity level. ICMB 92888, ICMB 92111, ICMR 13666, ICMA4 03111, ICMA1 18888, and ICMA1 11999 displayed a high level of genetic relatedness, as the dissimilarity coefficient was the lowest among other pairs. Based on the frequency of occurrence, ICMB 101791 and ICMA1 1803 (within B-lines) and ICMR 100258 and ICMR 16333 (within R-lines) were the genotypes showing a high level of genetic diversity with the other genotypes in their respective groups.

**Figure 4:**
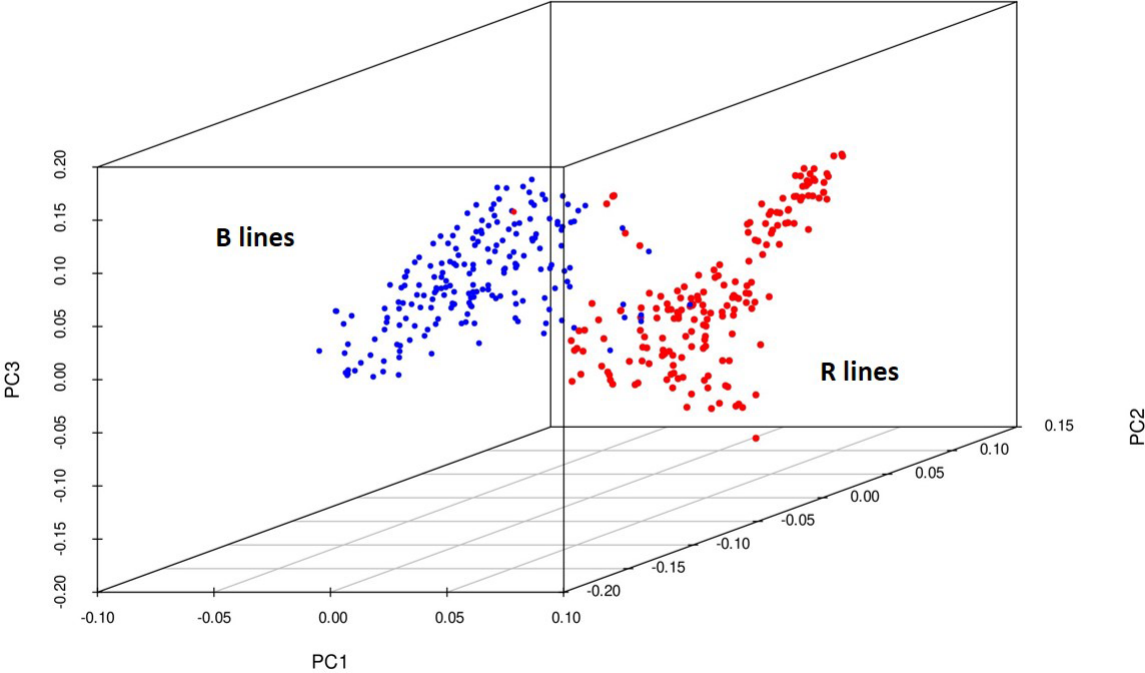
3D plot of the principal components from 4K SNP data set explaining a clear-cut separation B- and R-line groups of pearl millet.

Principal components were generated for the 373 genotypes using the function SnpgdsPCA available in the SNPRelate R package. The percentage of variation calculated for the first 15 principal components was 34%, and the three-dimensional plotting of genotypes was done for the first three components (**Figure 4**) to determine the grouping pattern of the genotypes. The PCA grouped all the genotypes into two major groups, namely, B and R lines, according to their pedigree which agreed with the genetic diversity model results.

**Figure 5:**
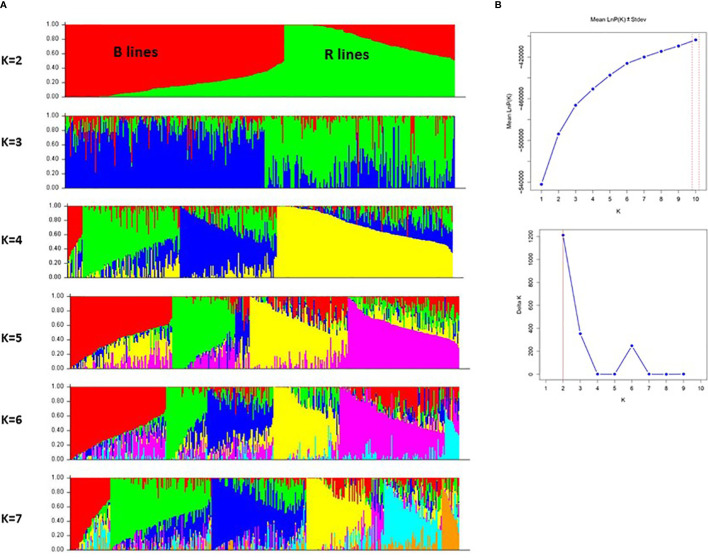
**(A)** A graphical display of the genetic structure of 373 pearl millet inbreds at K value 2 to 7 forming different clusters and exhibiting different levels of admixture. **(B)** Population structure analysis with mean LnP(K) and delta K values, showing the best assumed groups for the given population is 2.

We further investigated the presence of population structure in the 373 genotypes using Structure v2.3.4. The result showed that using mean LnP(K) and delta K values, population structure analysis reveals that the best-assumed group for the current population is 2 (**Figure 5**). The first sub-population (red color) had 193 genotypes, of which 185 were B-lines and eight were R-lines. A total of 184 genotypes, including 179 R-lines and five B-lines, made up the second sub-population cluster (green color). The genotypes of the B-line cluster (182) and the R-line cluster (108) lie in the > (70-90%) allele frequency range. About 23% of genotype accessions from both population clusters showed some admixtures. The structure model separated the whole population according to the pedigree and matched with the results of GD and PCA models.

### Genetic variation among and within group of accessions

AMOVA was performed to estimate the genetic differentiation of populations within and between B- and R-line groups. The results showed that a significant difference was available between the B- and R-line groups. The variation among and within the B- and R-line groups accounted for 13% and 87% of the total variation, respectively. The estimated pairwise PhiPT (Analog of fixation index FST) value between the B- and R-line groups was 0.13. (**Table 2**).

**Table 2:** Analysis of molecular variance of 4K SNPs for the 195 B-lines and 182 R-lines of pearl millet.

Data set	Source	df	SS	MS	Est. Var.	Variance (%)	PhiPT	Significant
4K SNPs	Among groups	1	13226.23	13226.23	67.87	13	0.132	0.001
	Within groups	375	168008.8	448.02	448.02	87		
	Total	376	181235.1		515.89	100		

df- degrees of freedom, SS- Sum of Squares, MS- Mean square, Est.Var- Variance Estimate, PhiPT- Analog of fixation index (Fst).

### Linkage disequilibrium

**Figure 6:**
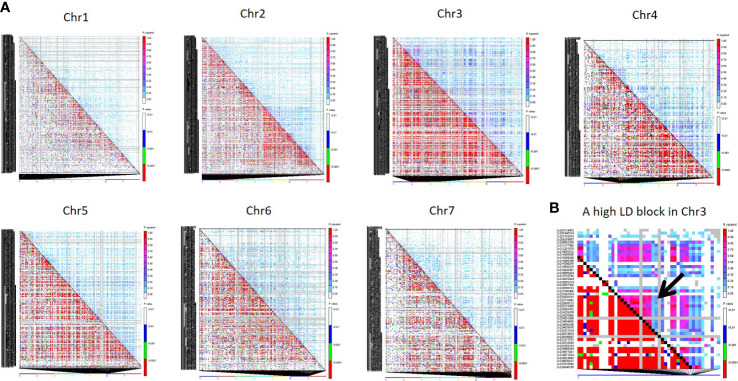
**(A)** Triangle plot of all seven chromosomes representing pairwise LD among all 4K SNPs. Pairwise LD values are plotted on both the X- and Y-axes; above the diagonal displays squared correlation coefficient (*r* ^2^) value and below the diagonal displays the corresponding P-values. **(B)** The LD plot of a Chr3 region displays a high LD pattern in a distance of 6.3 Kb to 1.5 Mb for 7 SNP pairs.

The *r2* was used to estimate LD between all SNPs of the 4K set on each chromosome through TASSEL 5 (Bradbury et al., 2007). Among seven chromosomes, Chr3 showed the highest LD, followed by Chr4, Chr5, and Chr6, while Chr7 showed the lowest LD (**Figure 6**).

Based on the *r^2^* value, the LD level was classified as high (0.90-1.00), moderate (0.50-0.90), low (0.10-0.50), and very low (0.00-0.01). Around 278 pairwise SNP were classified as high LD (*r^2^* >0.90) across the genome. Around 113 SNP pairs showed high LD (0.90-1) at chr3, followed by Chr2, and chr6, which had 47 and 35 high LD SNP pairs, respectively. Clusters of SNP pairs in high LD were primarily found in Chr3, followed by Chr2 and Chr6. A high LD block spanning the length of ~1.5 Mb in the middle of Chr3 (**Figure 6**) where three SNP pairs covered 1.5Mb distance and 4 SNP pairs covered 291kb. Chr1 had 14 pairs of SNPs between 0.90 - 1 *r^2^* range, the least among all chromosomes. The length of high LD regions (*r^2^* ≥ 0.90) ranged from 3 bp to ~117 Mb. More than 2000 SNP pairs were observed in a moderate LD range (0.50 to 0.90). Of this, Chr3 had the highest pairs (1123) followed by Chr4 (349) and Chr2 (248). The length of the LD blocks was identified in the range of ~27 bp to ~150.93 Mb under the moderate LD category. Around 28K pairs of SNPs have demonstrated low LD (0.10-0.50). Among all, the highest number of pairs was found in Chr3 (15,154 pairs) followed by Chr2 (8,916 pairs) and Chr5 (7037 pairs). The length of the genomic region in the low LD range was extended from 1 Mb to ~300 Mb. More than 167K pairs of SNPs showed a very low LD of <0.10. Chr2 had a high number of LD pairs (~33K), followed by Chr1 (29K) and Chr4 (17K) under the low LD class.

## Discussion

Plant breeding dynamics have changed since the 1980s with the development of molecular marker technologies (Hamrick, 1989). Marker-based genetic map facilitates plant genetics and breeding programs that bear the information for desired genes, alleles, or haplotypes. In pearl millet, Liu et al. (1994) created the first genetic map based on 181 RFLP markers. Later, (Qi et al., 2004) identified 353 RFLPs and 65 SSRs for linkage mapping. Further, other linkage maps were developed using EST-SSRs and DArT markers (Senthilvel et al., 2008; Supriya et al., 2011). These linkage maps suffer a high degree of marker clustering and lack uniform coverage.

Over the last decade, SNPs have been generated in several crops and have become the most popular genetic marker in trait mapping, molecular breeding, and population genetics experiments. The real explanation for SNPs becoming the marker of choice is their high abundance, even distribution in coding and non-coding regions, and the bi-allelic nature corresponding to the models studied in population genetics and co-dominant mode of inheritance (Khlestkina and Salina, 2006). Additionally, SNP genotyping offers logistical advantages such as a lower rate of genotyping error and greater ease of automating large-scale genotyping (Elshire et al., 2011).

Recent advances in NGS technologies have transformed the pace and precision of plant genomics, providing low-cost genotyping platforms that enable SNPs to be more readily used (Ganal et al., 2012). GBS is an NGS-based SNP detection method to perceive genome-wide SNPs and perform genotyping studies (Elshire et al., 2011). GBS technology provides large data volumes to a range of agronomically essential crops at a low cost per data point, irrespective of previous knowledge of genetic information, genome size, or ploidy information (Scheben et al., 2018).

SNP genotyping with precise sample tracking, collection, and DNA extraction is a potent tool to reshape breeding programs and increase selection gain (Chen et al., 2002). Despite the advantage of having less bias, the high-density SNP genotyping platforms require a sophisticated and complex pipeline with a deep understanding of bioinformatics to analyze the data, which limits the applicability of NGS in many breeding programs (Chen et al., 2014). On the other hand, the mid-density SNP markers are adequate for many breeding experiments at substantially lower cost and complication. Using SNP markers, a 1K RiCA mid-density panel was developed in rice (Arbelaez et al., 2019). Mid-density markers from DArTAG platform were available in maize (3305 SNPs), pigeon pea (2000 SNPs), wheat (3900 SNPs), common bean (1861 SNPs), groundnut (2500 SNPs), cowpea (2602 SNPs), and potato (2147 SNPs) (https://excellenceinbreeding.org). In pearl millet, the *de-novo* genome sequencing, followed by reference-based sequencing of 925 accessions (Varshney et al., 2017), paves the way for developing new SNP tools. Among different SNP densities, a medium density is a worthy addition to the genomic toolbox in pearl millet. Since there is no medium-density genotyping platform available in pearl millet, we developed a viable, cost-effective, and robust 4K SNP panel through “AgriSeq” genotyping technology and demonstrated its functional utility in genetic studies using 373 B and R lines.

### The SNP markers overview

The newly developed two 2K sets of SNPs, namely sets I and II, identified independently from the whole-genome sequences PMiGAP panel (345 genotypes) and breeding lines (580 genotypes), respectively, were distributed uniformly across the pearl millet chromosomes (**Figure 2A**). The gene ontology results showed that 2004, 1646, and 968 SNPs were associated with molecular function, biological process, and cellular component, respectively. Among various molecular functions, ATP binding was associated with more SNPs (318) followed by protein binding (235) and DNA binding (166) classes. More than 30 SNPs were associated with abiotic, oxidative, salt, and osmotic stresses under the biological process category. Nine SNPs identified in the cellular process category were related to heat shock responses. Membrane and nuclear-related functions were the top ones, as 38% of SNPs captured them from the cellular component.

The polymorphism information content (PIC) is one of the important measures to calibrate the informativeness of the marker. The higher value indicated that a marker has more alleles and can discriminate most individuals in a population (Botstein et al., 1980). Markers in our experiment had a PIC value as high as 0.38. The 2K set II had a high PIC (0.3) than 2K set I (0.16), while the combined 4K set had an average PIC of 0.23. The average expected heterozygosity (He) was higher (0.29) than the observed heterozygosity (Ho) (0.03) in the 4K set. The low level of observed heterozygosity was attributed to the fact that the inbreds attained almost homozygosity across loci, with minimal residual heterozygosity in the population. Among B- and R-lines, the observed heterozygosity in B-lines was higher than over R-lines. The selected marker panel genotyped through “AgriSeq” technology proved its utility as it discriminated the homozygotes and heterozygotes.

### Genetic diversity, PCA, and population structure

The pattern and degree of genetic diversity among 373 genotypes representing the gene pool of B and R groups were examined using 4K set SNPs. All genotypes were divided into two major groups based on the NJ analysis of the genetic dissimilarity coefficients. The B- and R- lines were further grouped into three subgroups, with some of the B-lines clustered with R-lines and *vice-versa* due to the fact that they share some level of parentage with the respective neighbors. The cross-grouping of B and R lines was also found in previous studies in pearl millet (Nepolean et al., 2012). Pairs with different levels of genetic dissimilarity were identified in both B and R groups. R groups had higher mean genetic diversity over B-lines. The higher gene diversity and the more alleles detected in R-lines were attributed to the broader genetic base of these lines while breeding these pollinator lines. The genetic distance among the lines will provide an opportunity to select precise crosses in heterosis breeding programs (Thirunavukkarasu et al., 2013). A set of highly genetically dissimilar pairs were identified within the B- and R-line pools. New B- and R-lines can be created by exploiting the genetic variability available in the respective pools (**Table 3**). Additionally, pairs with high genetic dissimilarity between B- and R-line pools were identified. These pairs can be used for generating heterotic combinations by exploiting the GCA and other beneficial agronomic traits (**Table 4**).

**Table 3:** Pairs of highly genetically dissimilar (>0.45) lines captured by the 4K SNP panel for use in developing new lines in respective B- and R-line pools.

S.No	B-lines		Genetic dissimilarity value	R-lines		Genetic dissimilarity value
	Line 1	Line 2		Line 1	Line 2	
1	ICMB 101791	ICMA1 101805	0.578	ICMR 100258	ICMR 07777	0.655
2	ICMA1 1803	ICMB 101564	0.545	ICMB 92888	ICMR 100258	0.592
3	ICMA1 101877	ICMA1 101813	0.504	ICMB 92111	ICMR 100258	0.580
4	ICMB 101578	ICMA1 1803	0.499	ICMR 100258	ICMR 19222	0.579
5	ICMA1 09222	ICMA1 101813	0.495	ICMR 102545	ICMR 16333	0.565
6	ICMA1 101813	ICMA1 101799	0.493	ICMR 100258	ICMR 1203	0.565
7	ICMB 101830	ICMA1 101813	0.492	ICMR 101859	ICMR 100258	0.563
8	ICMA1 101327	ICMB 101564	0.486	ICMR 16333	ICMR 14111	0.547
9	ICMB 101888	ICMB 101791	0.485	ICMB 92888	ICMR 16333	0.540
10	ICMB 101793	ICMB 101791	0.481	ICMR 102277	ICMR 14111	0.527
11	ICMB 101831	ICMB 101600	0.480	ICMR 11999	ICMR 07777	0.521
12	ICMB 101578	ICMA4 02111	0.476	ICMR 102283	ICMR 16333	0.520
13	ICMA5 02444	ICMA4 01444	0.476	ICMR 07777	ICMR 102539	0.520
14	ICMA1 18888	ICMB 101564	0.475	ICMR 18888	ICMR 16333	0.520
15	ICMB 101578	ICMA1 92777	0.475	ICMR 17111	ICMR 16333	0.518
16	ICMB 101879	ICMB 101564	0.473	ICMR 14111	ICMR 10888	0.514
17	ICMA1 101813	ICMA1 101811	0.473	ICMR 19888	ICMR 16333	0.514
18	ICMB 101878	ICMA1 101813	0.472	ICMR 16333	ICMR 101307	0.512
19	ICMA1 101805	ICMA1 101799	0.471	ICMB9 2111	ICMR 102277	0.511
20	ICMB 101832	ICMA1 101805	0.468	ICMR 13999	ICMR 07777	0.503
21	ICMB 101889	ICMB 101791	0.468	ICMR 19222	ICMR 16333	0.503
22	ICMA1 92777	ICMA1 100718	0.467	ICMR 09222	ICMR 07777	0.502
23	ICMB 101791	ICMB 101789	0.466	ICMR 102012	ICMR 16333	0.502
24	ICMB 101885	ICMA5 02444	0.464	ICMR 102547	ICMR 07777	0.502
25	ICMA1 19888	ICMB 101564	0.463	ICMR 16333	ICMR 102504	0.502

**Table 4:** Pairs of highly genetically dissimilar (>0.5) lines captured by the 4K SNP panel for use in developing new hybrid combinations.

S No	Potential crosses		Genetic dissimilarity Value
	B-Line	R-Line	
1	ICMA1 101813	ICMR 100258	0.687
2	ICMB 101791	ICMR 16333	0.606
3	ICMB 101791	ICMR 07777	0.595
4	ICMB 101791	ICMR 16777	0.553
5	ICMB 101791	ICMR 10888	0.568
6	ICMB 101791	ICMR 102497	0.563
7	ICMA5 02444	ICMR 102545	0.560
8	ICMB 101791	ICMR 14111	0.546
9	ICMB 101791	ICMR 16444	0.543
10	ICMB 101791	ICMR 102277	0.543
11	ICMB 101791	ICMR 10111	0.538
12	ICMB 101791	ICMR 100168	0.530
13	ICMA1 101813	ICMR 17333	0.530
14	ICMB 101791	ICMR 102151	0.530
15	ICMA1 101813	ICMR 102499	0.529
16	ICMA1 101813	ICMR 07111	0.529
17	ICMB 101791	ICMR 08888	0.526
18	ICMA1 101813	ICMR 07888	0.525
19	ICMA1 101813	ICMR 18111	0.524
20	ICMB 101791	ICMR 15333	0.524
21	ICMA1 101813	ICMR 06999	0.524
22	ICMB 101791	ICMR 12111	0.524
23	ICMA1 101813	ICMR 102548	0.523
24	ICMB 101791	ICMR 07999	0.522
25	ICMA1 101813	ICMR 15111	0.521

The number of subpopulations was validated by plotting the PCA of the genetic data. PCA captures the continuous axes of genetic variation by correlating and ranking the genotypes (Price et al., 2006). The PCA showed two major groups plotted in the first three axes. There is great diversity within these subgroups, as evidenced by the fact that the first 15 PCA components explained more than 34% of the variation and broader divergence of the lines. Population structure plays an integral part in understanding evolutionary genetics and illustrating the diversity of a population. In the present study, the structure model revealed that the 373 genotypes from diverse sources originated from two genetic populations (K= 2), which were expected as they belong to different B and R breeding groups. Population structure also revealed a smaller amount of admixture between the two populations, which explained that they share common breeding history. While developing the B and R lines, the lines from other groups might have been used to introduce new and valuable traits unavailable in the respective pools.

The grouping of B and R lines in our study can be traced back to the history of breeding in pearl millet. The B- and R-lines are named female lines and pollinator lines, respectively, in hybrid breeding. These two groups represent a putative heterotic genotype pool having favorable alleles for increasing the yield. In order to maintain the heterotic potential between B and R lines, line development programs strictly used the lines within respective groups, and new hybrids were generated using B and R line crosses. Hence, separate genetic pools have been maintained between two different populations. Our newly developed 4K SNP panel captured the genetic properties such as genetic diversity, PCA, and population structure of the B and R lines. The genetic relationship between and among B and R line groups will be helpful in developing new heterotic pools and segregating populations for trait mapping, and conducting association mapping and genomic selection experiments.

### Genetic variation among and within group of accessions

The B and R line groups, classified based on the pedigree, were analyzed to characterize the genetic differentiation between and within the subgroups using AMOVA. The relative contribution between populations to the overall genetic variation is described by phi-statistics (PhiPT), a modified form of Wright’s F(Fst). The genetic variation between individuals within a population and the population’s divergence from the Hardy-Weinberg proportions are measured by Fst (Wright et al., 1978). The AMOVA results of the current experiment showed that the majority of the variation within sub-populations accounted for 87% (P <0.001) of the total variation, and the between-population differences accounted for 13% (P <0.001) of the variation (**Table 2**). It indicated that a large part of the accessions within the groups showed a high-level genetic variability. Previously, a set of 213 old and 166 newly-generated pearl millet parental lines were genotyped by 28 SSRs, and the subsequent AMOVA analysis of the old and new sets showed that the genetic variation between B and R lines was 16.98% and 9.22%, respectively (Gupta et al., 2015).

This was further supported by the combined AMOVA of both sets, which showed a significant difference between the B- and R-line groups. A range of 0 to 0.05, 0.05 to 0.15, 0.15 to 0.25, and >0.25 indicate little, moderate, large, and great genetic differences, respectively (Wright et al., 1978). While comparing the genetic differentiation, rice showed PhiPT value of 0.130 between the *indica* and *japonica* groups (Luong et al., 2021), and the Ethiopian sorghum group showed 0.252 between B- and R-lines (Mindaye et al., 2015). Finger millet revealed a moderate genetic differentiation (Fst = 0.352) among seven population sub-groups (Brhane et al., 2022). Pearl millet demonstrated genetic differentiation at a PhiPT value of 0.130 for the chosen genotypes, similar to those studies. It also implied that the current marker set used in this experiment could extract the molecular variance at a population level and can be used for further applications such as designing crosses based on genetic diversity, developing mapping populations for trait mapping, and conducting genomic selection experiments.

### Linkage disequilibrium

The potential response to both natural and artificial selection is constrained by the non-random association of alleles at two or more loci, which also offers information about past events. LD reflects the history of natural selection, gene conversion, mutation, and other forces influencing gene frequency and evolution. LD provides insight into previous evolutionary events and explains the co-evolution of linked sets of genes. LD-based on Pearson correlations (*r^2^*) is a squared value of the correlation between pairs of markers across the genome. The details of the LD pattern are used in mapping genes associated with complex quantitative traits. In association studies, it has frequently been discovered that markers directly related to the mutation exhibit less LD than those more distantly related. The test of LD is crucial because it will help to quickly and efficiently choose the SNP markers that can be used for trait mapping and selection studies.

In our result, uniformly distributed markers showed the regions of high and low LD on various chromosomes of pearl millet (**Figure 6**). Comparing the high LD pairs on all chromosomes, Chr3 captured 40% high LD pairs, and the length of the high-LD pairs on Chr3 ranged from 1Mb to 117Mb. Chr3 is the longest one (346 Mb) among all chromosomes, so that it would have captured more LD events. The higher LD in Chr3 is attributed to the low level of recombination events and fixation of alleles. Among high LD (*r^2^* <0.90) SNP pairs, 63% were derived from PMiGAP, and 36% SNPs from breeding lines since the PMiGAP represented the accessions with greater genetic diversity over the cultivated breeding lines and was clearly captured by the newly developed 4K SNP panel. The knowledge of LD regions from the B and R lines of the pearl millet genomes characterized by the newly developed mid-density set provides the opportunity to exploit them in the genetic characterization of diverse germplasm, trait mapping, and genomic selection experiments.

## Conclusions

We have successfully identified and validated a mid-density marker set for routine genotyping of pearl millet lines and its usefulness for various genomic studies. By mining 925 pearl millet genomes comprising genetically diverse wild and breeding lines, a set of 4112 SNPs were identified. A panel of 373 B and R lines was genotyped by these SNPs using the Agri-Seq platform. The results showed that the newly developed SNPs were uniformly distributed across the genome and had significant PIC and gene diversity. The SNP panel was used to group the genotypes through diversity, PCA, and structure models. All three statistics showed consistent results where they separated the accessions into two major groups, B and R lines. The LD analysis showed the regions of high and low LD. The AMOVA revealed a significant distinction between the B- and R-line groups and the extent of genetic divergence within and across and R lines groups. This research demonstrated that pearl millet has a high degree of genetic diversity and variable levels of LD across the genome, which are highly beneficial in developing heterotic groups for hybrid breeding. The experiment revealed that our mid-density 4K SNP panel genotyped by AgriSeq technology had a high level of information, making them suitable for several uses, including trait mapping, marker-assisted backcrossing, and genomic selection for pearl millet improvement.

## Data availability statement

The original contributions presented in the study are included in the article/**Supplementary Material**. Further inquiries can be directed to the corresponding author.

## Author contributions

NT conceptualized the experiment. JS, SS, AR, AC, JI performed the data analysis. SKG, RM, MG, SR contributed and maintained the genotypes. KG, SH, AV performed the genotyping experiments. All authors contributed to the final manuscript. All authors read and approved the manuscript. All authors contributed to the article and approved the submitted version.
